# Correlation Between Urine Formaldehyde and Cognitive Abilities in the Clinical Spectrum of Alzheimer’s Disease

**DOI:** 10.3389/fnagi.2022.820385

**Published:** 2022-02-10

**Authors:** Ying Wang, Fengfeng Pan, Fang Xie, Rongqiao He, Qihao Guo

**Affiliations:** ^1^Department of Gerontology, Shanghai Jiao Tong University Affiliated Sixth People’s Hospital, Shanghai, China; ^2^PET Center, Huashan Hospital, Fudan University, Shanghai, China; ^3^State Key Laboratory of Brain and Cognitive Sciences, Institute of Biophysics, Chinese Academy of Sciences, Beijing, China; ^4^Key Laboratory of Mental Health, Institute of Psychology, Chinese Academy of Sciences, Beijing, China

**Keywords:** Alzhaimer’s disease, urine formaldehyde, biomarker, APOE, β-amylaoid

## Abstract

Urine-based formaldehyde has been reported to be a potential biomarker for Alzheimer’s disease (AD). However, there is a lack of research about the correlation between urine formaldehyde and cognitive abilities in the clinical spectrum of AD, especially the preclinical period. The relationship of urine formaldehyde with APOE genotype, brain Aβ status and plasma pathological markers in AD are also not clear. This study intends to explore the correlation between urine formaldehyde and cognitive abilities throughout the AD continuum, to evaluate the role of APOE genotype and Aβ accumulation on urine formaldehyde, and further to clarify the relationship between urine formaldehyde level and AD plasma pathological markers. We recruited 72 cognitively normal controls (NC), 110 subjective cognitive decline (SCD), 140 objectively defined subtle cognitive decline (Obj-SCD), 171 mild cognitive impairment (MCI) and 136 AD dementia participants. Next, we collected the data of clinical materials, neuropsychological examination, APOE genotyping, urine formaldehyde concentration, 18F-florbetapir PET imaging and plasma biomarkers. Compared with NC, Obj-SCD and MCI groups, the level of urine formaldehyde was found to be significantly upregulated in SCD group. In addition, the level of urine formaldehyde was significantly higher in AD group compared to both NC and MCI groups. Further subgroup analysis showed that, the level of urine formaldehyde was higher in APOE ε4+ subgroup compared to APOE ε4– subgroup in both NC and AD groups. There was no difference in urine formaldehyde level between the brain Aβ+ subgroup and Aβ– subgroup in each group. In addition, regression analysis showed urine formaldehyde level was correlated with gender, plasma Aβ42 and p-Tau181/T-tau. The dynamic change of urine formaldehyde in the AD continuum could be used as a potential biomarker, and combined with comprehensive cognitive evaluation could become a useful method to distinguish SCD from NC and Obj-SCD, and to distinguish MCI from AD.

## Introduction

Alzheimer’s disease (AD) is a neurodegenerative disease that is characterized by the deposition of extracellular amyloid plaques composed of highly aggregated amyloid beta (Aβ) peptides Aβ40 and Aβ42, as well as intracellular neurofibrillary tangles (NFTs) consisting of hyper-phosphorylated tau protein, progressive atrophy, and cognitive decline (Alzheimer’s Association, 2015; Knopman et al., 2021). The disease is now considered to fall along a continuum with long preclinical and prodromal stages, where pathophysiological change and potentially irreversible brain damage occur many years prior to the appearance of clinically apparent manifestation (Sperling et al., 2011; Jack et al., 2018). Based on the degree of cognitive impairment, AD is often divided into three stages: The preclinical stage, including subjective cognitive decline (SCD) and objectively defined subtle cognitive decline (Obj-SCD), the prodromal stage, characterized by mild cognitive impairment (MCI), and the actual dementia stage with functional impairment (Albert et al., 2011; Sperling et al., 2011; Jack et al., 2018; Thomas et al., 2020). SCD refers to individuals who perceive themselves as experiencing a decline in cognitive functioning in the absence of objective findings on neuropsychological testing. Obj-SCD refers to those whose cognitive change can be captured during the preclinical phase of AD using sensitive neuropsychological measures. These two have been shown to be risk factors and very early symptoms of later cognitive decline and dementia (Koppara et al., 2015; Thomas et al., 2020).

The repeated failure of clinical trials in drug development and the absence of effective treatments for AD have resulted in a surge of research focusing on the early diagnosis and intervention. As efforts to detect a disease earlier in the continuum and to assess the disease more accurately, the use of biomarkers is becoming a central facet of accurate diagnosis. Currently, the optimal biomarkers used to detect AD processes early in the course of the disease are pathological marker Aβ and tau by Positron Emission Tomography (PET) imaging or cerebrospinal fluid detection (CSF) (Lista et al., 2014; Jack et al., 2018). However, PET imaging is not universally available and obtaining CSF is a relatively invasive procedure. Attempt has been made to supplement these relatively specific biomarkers with other biomarkers that might be more applicable to large scale preliminary screening, early diagnosis and therapeutic effect monitoring in population. For example, there are a few studies examining the potential of urine as a biomarker fluid in AD (Zengi et al., 2011; Wang et al., 2015; Tong et al., 2017). Urine is a complex fluid with metabolites that reflect a response to injury (Lindsay and Costello, 2017) and oxidative stress (Terrill et al., 2020) among other biological events at the systems level and might, therefore, be useful as a target fluid in neurodegeneration and other brain diseases (An and Gao, 2015).

Urine-based formaldehyde has been reported to be a potential biomarker for AD (Tong et al., 2017). In humans, endogenous formaldehyde is typically present in the brain, blood, urine, and other tissues (Yu et al., 2003). Compared with brain and serum samples, urine formaldehyde measurement is a non-invasive and widely available method with the presence of fewer interfering proteins. The concentration of endogenous formaldehyde tends toward equilibrium (around 0.083 mmol L^–1^ in urine) under normal physiological conditions, but becomes unbalanced under certain stresses. Urine formaldehyde level increases during aging and also increases in AD patients (He et al., 2010; Tong et al., 2017). According to the “formaldehyde stress” hypothesis based on basic research, the abnormal accumulation of endogenous formaldehyde can cause abnormal change in proteins, resulting in neuronal responses such as tau hyper-phosphorylation (Lu J. et al., 2013), DNA damage (Lu Y. et al., 2013), reduced long-term potentiation (Tong et al., 2013a) and even cell death (Szende and Tyihák, 2010), followed by associated dysfunctions and neurodegenerative diseases (Chen et al., 2006; Tong et al., 2011, 2013b; Wang et al., 2012). Several clinical studies suggest that urine formaldehyde concentration is significantly higher in patients with AD than in unaffected older adults (Tong et al., 2011, 2017). And the hippocampal formaldehyde concentration in AD patients is significantly higher than that in age-matched controls or young people in an autopsy study (Tong et al., 2013b). However, there is a lack research about the correlation between urine formaldehyde and cognitive abilities in the clinical spectrum of AD, especially the preclinical period.

The ε4 allele of apolipoprotein (APOE) is the most important genetic risk factor for AD occurring after 65 years with a two to threefold increased risk for AD in heterozygotes rising to about 12-fold in homozygotes (Corder et al., 1993). The presence of APOE ε4 allele is associated with greater amyloid deposition (Drzezga et al., 2009; Morris et al., 2010), alterations in brain function and glucose metabolism (Langbaum et al., 2009), as well as decreased level of Aβ42 in the CSF in patients with late MCI and AD and older adults with normal cognition (Vemuri et al., 2010; Risacher et al., 2013). However, it is not clear whether urine formaldehyde concentration is correlated with APOE genotype. The Aβ peptide has long been considered as the driving force behind AD and emerging data suggest that amyloid plaque accumulation is associated with brain change in AD patients. Therefore, it is vital to understand the relationship of brain Aβ deposition with urine formaldehyde.

Plasma biomarkers, including tau, neurofilament light chain (NfL) and Aβ are increasingly being used to define and stage Alzheimer’s disease (Ikram et al., 2020). Previous studies have documented that decreases in plasma Aβ42 and Aβ42/Aβ40 ratios variably associated with PET Aβ deposition (Nakamura et al., 2018). In addition, plasma phosphorylated tau 181 (P-tau181) strongly associates with PET Aβ load and differentiates AD from non-AD neurodegenerative diseases (Karikari et al., 2020); whereas total tau (T-tau) measures were slightly increased in AD in some studies (Pase et al., 2019). In parallel, elevated plasma level of NfL were found to be associated with neurodegeneration in AD patients (Mattsson et al., 2017). So far, it is not clear whether there is a correlation between these blood markers and urine formaldehyde in AD spectrum.

To date, there is limited data for change of urine formaldehyde in the AD clinical spectrum. And the correlation between urine formaldehyde and cognitive abilities in the clinical spectrum of AD, especially the preclinical period is still not clear. In this study, we aim to describe the change of urine formaldehyde that may be observed during the clinical progression from cognitively healthy stage to Alzheimer’s disease dementia and provide further insights into the correlation between urine formaldehyde and cognitive abilities throughout the AD continuum. We also intend to evaluate the role of APOE genotype and Aβ accumulation on urine formaldehyde, and further to clarify the relationship between urine formaldehyde level and AD plasma pathological markers.

## Materials and Methods

### Participants

A total of 629 participants from Sixth People’s Hospital, Shanghai, China, were recruited through the outpatient memory clinic and advertisements between January 2020 and December 2020. The participants were classified into five groups according to the clinical diagnosis, including 72 cognitively normal controls (NC), 110 SCD, 140 Obj-SCD, 171 MCI and 136 AD dementia participants. The patients with SCD were classified on the basis of their performance on the neuropsychological tests using the guidelines of Jessen et al. (2014). For Obj-SCD we used Jak/Bondi and coworkers’ criteria (Thomas et al., 2018). Patients with MCI were diagnosed based on an actuarial neuropsychological method proposed by Jak/Bondi as previously described (Bondi et al., 2014; Pan et al., 2021). Those patients with AD were diagnosed according to the National Institute on Aging-Alzheimer’s Association (NIA-AA) criteria (Petersen, 2004; McKhann et al., 2011). The following inclusion criteria have been followed: Aged 50–80 years, had completed at least 6 years of education, were fluent in Chinese, and had normal vision and hearing to complete cognitive tests. The following exclusion criteria were implemented: a history of significant neurologic disease and psychiatric disorders other than AD, together with metabolic disorders, nutritional deficiencies and infectious diseases which might influence one’s cognition, other potential causes of cognitive decline, such as cerebral infarction, subdural hematomas, hydrocephalus, intracranial tumors and infections by routine cranial MRI scanning, alcohol or other substances abuse.

This study was approved by the Ethical Committee for Medical Research of the Shanghai Jiao Tong University Affiliated Sixth People’s Hospital. The Written informed consent was obtained from each subject after the aims and protocol was fully explained.

### Clinical Assessments

A detailed clinical materials assessment and neuropsychological examination for all participants was performed as previously described (Cui et al., 2021; Pan et al., 2021). Briefly, all the participants underwent the cognitive screening tests of Mini-Mental State Exam (MMSE) and Montreal Cognitive Assessment—Basic (MoCA-B), a battery of standardized neuropsychological tests of memory, language, attention, executive function, and visuospatial ability. All the neuropsychological assessments were carried out in Mandarin Chinese by trained raters.

### Apolipoprotein Genotyping

The genomic DNA was extracted from whole-blood samples with the Spin Columns DNA Isolation Kit (Generay Biotech Co., Ltd., Shanghai, CN) following the manufacturer’s protocol. Two polymorphic sites, rs. 429358 and rs. 7412, consisted of an APOE genotype, were determined by a ligase detection reaction (LDR)–fluorescent nanosphere technique (NEB Company, United States) according to the manufacturer’s instructions. Briefly, multiplex ligase detection reaction amplification was performed using fluorescently labeled magnetic nanosphere-bound upstream LDR probes and downstream probes labeled with a unique fluorescent group for each SNP locus. The amplified LDR products were separated by magnetic nanospheres and then scanned by fluorescence spectroscopy.

### 18F-Florbetapir Positron Emission Tomography Acquisition and Analysis

Some participants underwent an 18F-florbetapir PET scanning using PET/CT system (Biograph mCT Flow PET/CT, Siemens, Erlangen, Germany) within 1 month at the PET center of Huashan hospital, Fudan University. Briefly, subjects were intravenously injected with dose of about 10 mCi (370 MBq) of 18F-AV45 and rested quietly for 50 min. Then, a 20 min PET acquisition was performed with a low-dose CT scanning. After acquisition, the PET images were reconstructed by filtered back projection algorithm with corrections for decay, normalization, dead time, photon attenuation, scatter and random coincidences. For 18F-florbetapir-PET image interpretation, positive and negative amyloid deposition was determined visually by 3 physicians independently and blind to clinical diagnosis.

### Analysis of Urine Formaldehyde by High Pressure Liquid Chromatography

The morning urine samples were collected in the same week after the neuropsychological tests. The urine samples were first centrifuged at 4°C at 12,000 rpm for 10 min, and then 0.4 mL urine supernatants were collected, followed by mixing with 2, 4-dinitrophenylhydrazine (DNPH, final concentration 0.1 g/L in acetonitrile) and 0.1 mL trichloroacetic acid. The samples were vortexed for 30 s and then centrifuged at 4°C at 12,000 rpm for 10 min. The supernatants were transferred to a 2 mL glass vial, heated in a 60°C water bath for 30 min, and analyzed using HPLC system (LC-20A, Shimadzu, Japan) with an ultraviolet detector. The mobile phase was 65% acetonitrile in water, with a flow rate of 0.8 mL/min and column temperature of 35°C. The formaldehyde-DNPH derivative was eluted from the HPLC column at a retention time of 6–7 min at the maximum wavelength of 355 nm.

### Blood Biomarker Measurements

The blood was centrifuged, aliquoted, and stored at −80°C until use. All biomarkers were measured on the Single Molecular Array (Simoa) HD-1 Analyzer platform (Quanterix, Billerica, MA, United States) according to the manufacturer’s instructions (Chong et al., 2021). The AT270 mouse monoclonal antibody specific for the threonine-181 phosphorylation site was used to measure the concentration of plasma phosphorylated P-tau181 based on an ultrasensitive Simoa immunoassay. Plasma Aβ40, Aβ42, and T-tau were measured using the Neurology 3-plex A assay kit from Quanterix. NfLwas measured by the Simoa NF-light VR advantage kit.

### Statistical Analysis

One-way analysis of variance (ANOVA) was used to assess the mean differences in the age, education, scores on the cognitive and neuropsychological tests and urine formaldehyde concentration among the different diagnostic groups, and the *post hoc* tests were conducted by LSD multiple comparison tests. Student’s *t*-test was used for continuous value between ε4+ subjects and ε4- subjects, as well as Aβ+ subjects and Aβ- subjects of each diagnostic group. The differences between rates were tested by χ^2^ or Fisher exact tests, if appropriate. For all quantitative data, results are expressed as the mean ± standard deviation. Correlations between urine formaldehyde level and cognition scores or plasma biomarkers were assessed using the Spearman correlation coefficient. Moreover, multiple linear regression analysis was used to exclude the influence of covariates on the correlation of urine formaldehyde level. *p* < 0.05 was considered significant. All analyses were performed with Statistical Package for the Social Sciences 22.0 Software (SPSS 22.0).

## Results

### Demographic and Clinical Characteristics of Diagnostic Groups

A total of 629 subjects were enrolled in the current study and divided into five diagnostic groups, including NC, SCD, Obj-SCD, MCI, and AD dementia. All participants underwent urine formaldehyde detection.

The detailed descriptive statistics for basic demographic and clinical characteristics among five diagnostic groups were shown in Table 1. There were no significant differences in age and gender. Consistent with literature reports, the education years, proportion of APOE ε4 carriers and neuropsychological test performance was significantly different among five diagnostic groups (*P* < 0.001), with lower educational attainment, more serious cognitive impairment and a higher APOE ε4 carriers’ positive rate in AD groups compared to NC group (*P* < 0.05).

**TABLE 1:** Demographics, disease characteristics and urine formaldehyde of five diagnostic groups.

	NC	SCD	Obj-SCD	MCI	AD	*P*-value	*P*-value	*P*-value	*P*-value	*P*-value
	(*n* = 72)	(*n* = 110)	(*n* = 140)	(*n* = 171)	(*n* = 136)	(five groups)	(NC vs. SCD)	(SCD vs. Obj-SCD)	(SCD vs. MCI)	(MCI vs. AD)
Age (years)	65.5 ± 6.9	65.2 ± 6.3	66.2 ± 6.5	67.0 ± 6.7	67.3 ± 7.6	0.078^a^	–	–	–	–
Male (%)	28 (39%)	36 (33%)	49 (35%)	57 (33%)	48 (35%)	0.923^c^	–	–	–	–
Education (years)	12.3 ± 2.8	12.1. ± 2.8	11.7. ± 2.9	11.0. ± 2.7	9.5. ± 3.1	0.000^a^	0.649^b^	0.239^b^	0.001^b^	0.000^b^
APOE ε4 carriers (%)	18 (25%)	27 (25%)	25 (18%)	42 (25%)	65 (48%)	0.000^c^	1.000^c^	0.212^c^	1.00^c^	0.000^c^
MMSE	28.5 ± 1.3	28.1 ± 1.5	27.4 ± 1.7	26.6 ± 1.8	15.9 ± 5.5	0.000^a^	0.364^b^	0.076^b^	0.029^b^	0.000^b^
MoCA-B	26.3 ± 2.2	25.0 ± 3.9	23.9 ± 3.4	21.5 ± 3.2	10.9 ± 5.3	0.000^a^	0.022^b^	0.030^b^	0.000^b^	0.000^b^
Urine formaldehyde (μg/ml)	8.6 ± 3.9	10.2 ± 4.9	9.1 ± 4.2	9.0 ± 4.1	11.0 ± 4.9	0.000^a^	0.014^b^	0.046^b^	0.029^b^	0.000^b^

*MMSE, Mini-Mental State Exam; MoCA-B, Montreal Cognitive Assessment—Basic.*

*Data were shown as mean ± standard deviation (range) except where indicated.*

*^a^One way ANOVA.*

*^b^Post-hoc tests were conducted by LSD multiple comparison test.*

*^c^χ^2^-test.*

### Urine Formaldehyde Concentration Is Correlated With Cognitive Abilities in the Clinical Spectrum of Alzheimer’s Disease

**FIGURE 1:**
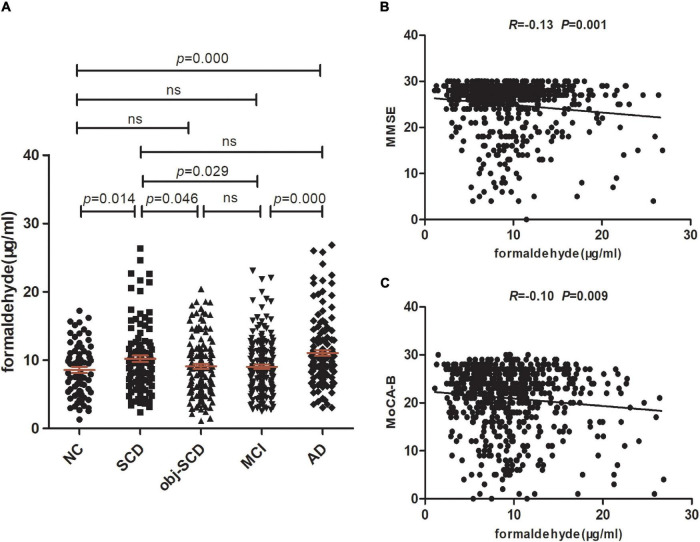
Urine formaldehyde concentration is correlated with cognitive abilities in the clinical spectrum of AD. **(A)** Urine formaldehyde level in five diagnosis groups. **(B)** The correlations between urine formaldehyde and MMSE. **(C)** The correlations between urine formaldehyde and MoCA-B.

In the clinical spectrum of Alzheimer’s disease, significant effects of diagnosis on the urine formaldehyde were observed (*P* < 0.001). The level of urine formaldehyde presented a trend of firstly increasing in SCD, then decreasing in Obj-SCD, and finally increasing in MCI and AD period (Table 1 and Figure 1A). To be specific, compared with NC (*P* < 0.05), Obj-SCD (*P* < 0.05) and MCI (*P* < 0.05) groups, the level of urine formaldehyde was found to be significantly upregulated in SCD group. There was no significant difference between SCD group and AD group. In addition, the level of urine formaldehyde was significantly higher in AD group compared to both NC (*P* < 0.001) and MCI (*P* < 0.001) groups. However, no significant difference in urine formaldehyde level was observed among NC, Obj-SCD and MCI groups. After adjustment for education years, cognitive scores, percentage of APOE ε4 carriers by covariance analysis, the difference of urine formaldehyde level among the five groups still exists (*P* < 0.001).

We next estimate the relationship between formaldehyde level in the urine and general cognitive ability of 629 participants. We found that urine formaldehyde concentration were inversely correlated with the MMSE scores (*R* = −0.13, *P* < 0.01) and MoCA-B scores (*R* = −0.10, *P* < 0.01) (Figures 1B,C).

### The Role of Apolipoprotein Genotype on Urine Formaldehyde in Each Diagnostic Group

In the present study, we sought to evaluate the impact of the presence of APOE ε4 alleles on urine formaldehyde concentration in each diagnostic group. All participants underwent APOE genotyping and then were further categorized by APOE ε4 status in each diagnostic group, consisting of APOE ε4+ participants with one or more ε4 allele (ε2/ε4, ε3/ε4, ε4/ε4) and APOE ε4- participants without an ε4 allele (ε2/ε2, ε2/ε3, ε3/ε3).

**FIGURE 2:**
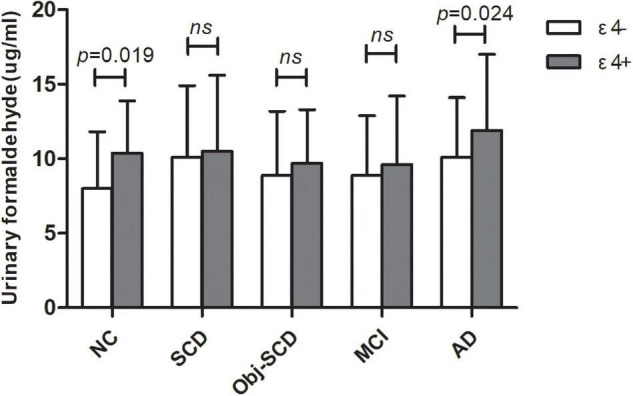
The role of APOE genotype on urine formaldehyde in each diagnostic group. Urine formaldehyde level was analyzed according to APOE ε4 genotype in each diagnosis group.

The clinical details of ε4 positives and negatives in the five groups were shown in Table 2. There were no statistic difference in age, gender, education, MMSE and MoCA-B between ε4– and ε4+ subgroups in each group. We found that there was a significant difference in urine formaldehyde level between APOE ε4+ and APOE ε4- subjects in NC and AD groups. Compared to APOE ε4- subgroup, the level of urine formaldehyde was higher in APOE ε4+ subgroup in both NC and AD groups (Table 2 and Figure 2). Differences were not found in the remaining groups, including SCD, Obj-SCD and MCI groups (Table 2 and Figure 2). There was a significant impact of the APOE ε4 presence on urine formaldehyde level in NC and AD groups.

**TABLE 2:** Demographics, disease characteristics and urine formaldehyde of ε4– and ε4+ participants in five diagnostic groups.

Groups	APOE genotype	Age (years)^a^	Male (%)^b^	Education (years)^a^	MMSE^a^	MoCA-B^a^	Urine formaldehyde (μg/ml)^a^
NC	ε4–(*n* = 54)	64.9 ± 6.5	19 (35)	12.6 ± 2.8	28.5 ± 1.3	26.4 ± 2.3	8.0 ± 3.8
	ε4+(*n* = 18)	67.4 ± 7.6	9 (50)	11.6 ± 3.0	28.6 ± 1.3	26.1 ± 2.0	10.4 ± 3.5
	*p*-value	0.179	0.280	0.196	0.721	0.584	0.019

SCD	ε4–(*n* = 83)	65.5 ± 6.0	29 (35)	12.1 ± 2.8	28.2 ± 1.3	25.3 ± 3.0	10.1 ± 4.8
	ε4+(*n* = 27)	64.3 ± 7.2	7 (26)	12.1 ± 3.0	27.9 ± 1.9	24.1 ± 5.8	10.5 ± 5.1
	*p*-value	0.397	0.482	0.980	0.374	0.168	0.747

Obj-SCD	ε4–(*n* = 115)	66.4 ± 6.2	41 (36)	11.7 ± 2.9	27.4 ± 1.7	24.0 ± 3.6	8.9 ± 4.3
	ε4+ (*n* = 25)	65.7 ± 7.9	8 (32)	11.6 ± 2.8	27.6 ± 1.5	23.6 ± 2.4	9.7 ± 3.6
	*p*-value	0.643	0.820	0.777	0.703	0.623	0.412

MCI	ε4–(*n* = 129)	66.6 ± 6.8	44 (34)	11.1 ± 2.8	26.7 ± 1.7	21.7 ± 3.0	8.9 ± 4. 0
	ε4+ (*n* = 42)	68.2 ± 6.4	13 (31)	10.7 ± 2.5	26.1 ± 1.7	20.6 ± 3.5	9.6 ± 4.6
	*p*-value	0.190	0.710	0.373	0.077	0.052	0.343

AD	ε4–(*n* = 71)	66.9.3 ± 7.8	25 (35)	9.5 ± 3.0	16.7 ± 5.4	11.9 ± 5.3	10.1 ± 4.0
	ε4+ (*n* = 65)	67.8 ± 7.4	22 (34)	9.5 ± 3.1	15.0 ± 5.6	10.1 ± 5.0	11.9 ± 5.1
	*p*-value	0.537	0.858	0.899	0.069	0.052	0.024

*MMSE, Mini-Mental State Exam; MoCA-B, Montreal Cognitive Assessment—Basic.*

*^a^Student’s t-test or Mann-Whitney U-test.*

*^b^χ^2^-test.*

### The Role of Brain Aβ Accumulation on Urine Formaldehyde in Each Diagnostic Group

**FIGURE 3:**
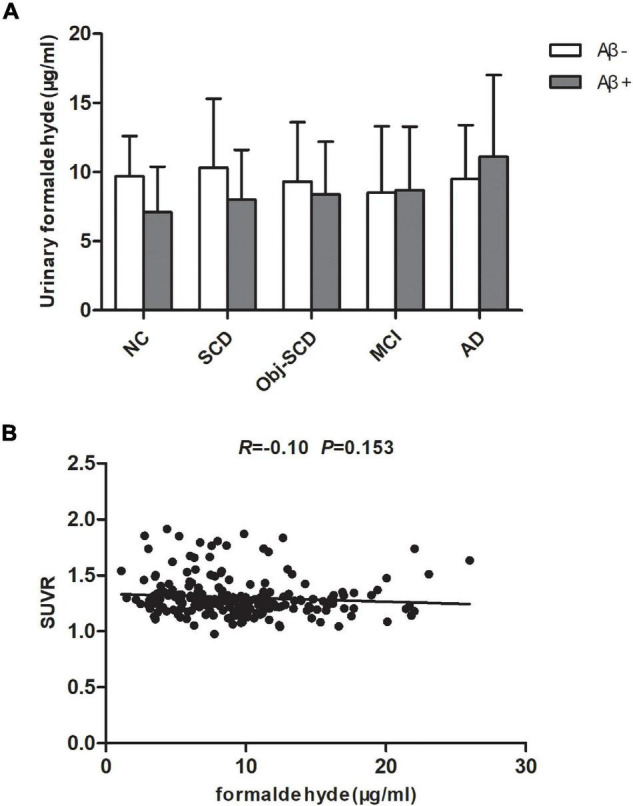
The role of brain Aβ accumulation on urine formaldehyde in each diagnostic group. **(A)** Urine formaldehyde level was analyzed according to brian amyloid status genotype in each diagnosis group. **(B)** The correlations between urine formaldehyde and SUVR.

As participants are heterogeneous as regard to their amyloid status, we further investigate the effect of Aβ deposition on urine formaldehyde. Participants in each diagnostic group were further classified as Aβ positive or Aβ negative according to 18F-florbetapir PET image results. Of the participants, only 237 completed 18F-florbetapir PET imaging. No systematic demographic differences between Aβ positive and Aβ negative participants in all the five groups were observed (Table 3). There was no difference in urine formaldehyde level between the Aβ+ and Aβ- subjects in each group. However, in the Aβ+ groups, urine formaldehyde was higher in the advanced stage compared with the previous stage of AD, although the difference was not statistically significant (Table 3 and Figure 3A). There was no significant impact of brain Aβ deposition on urine formaldehyde level in all diagnostic groups.

**TABLE 3:** Demographics, disease characteristics and urine formaldehyde of Aβ- and Aβ+ participants in five diagnostic groups.

Groups	Aβ status	Age (years)^a^	Male (%)^b^	Education (years)^a^	MMSE^a^	MoCA-B^a^	Urine formaldehyde (μg/ml)^a^
NC	Aβ– (*n* = 15)	66.2 ± 4.7	6 (40)	12.1 ± 2.8	28.1 ± 1.3	25.9 ± 3.6	9.7 ± 2.9
	Aβ+ (*n* = 8)	68.0 ± 5.8	4 (50)	11.7 ± 1.9	28.6 ± 1.6	26.4 ± 1.3	7.1 ± 3.3
	*p*-value	0.429	0.685	0.735	0.371	0.673	0.067

SCD	Aβ– (*n* = 34)	63.2 ± 6.3	12 (35)	12.1 ± 3.1	28.1 ± 1.6	25.5 ± 2.9	10.3 ± 5.0
	Aβ+ (*n* = 12)	66.7 ± 4.7	4 (33)	12.3 ± 1.9	28.9 ± 1.1	25.9 ± 2.1	8.0 ± 3.6
	*p*-value	0.091	1.000	0.861	0.098	0.630	0.142

Obj-SCD	Aβ– (*n* = 34)	64.6 ± 6.0	11 (32)	11.8 ± 2.5	27.6 ± 1.6	24.3 ± 3.1	9.3 ± 4.3
	Aβ+ (*n* = 20)	66.5 ± 5.1	9 (45)	13.2 ± 2.7	27.5 ± 1.5	24.4 ± 3.2	8.4 ± 3.8
	*p*-value	0.220	0.393	0.079	0.894	0.923	0.407

MCI	Aβ– (*n* = 41)	64.9 ± 5.4	17 (41)	11.0 ± 2.7	26.4 ± 1.9	21.6 ± 3.0	8.5 ± 4.8
	Aβ+ (*n* = 27)	66.5 ± 7.7	10 (37)	12.1 ± 2.5	26.3 ± 1.8	21.8 ± 3.3	8.7 ± 4.6
	*p*-value	0.309	0.803	0.105	0.755	0.778	0.877

AD	Aβ– –(*n* = 9)	64.6 ± 4.3	4 (44)	7.7 ± 2.2	17.2 ± 7.0	12.0 ± 5.5	9.5 ± 3.9
	Aβ+ (*n* = 37)	62.3 ± 6.6	14 (38)	9.1 ± 2.9	16.6 ± 5.1	11.9 ± 5.1	11.1 ± 5.9
	*p*-value	0.351	0.721	0.166	0.749	0.955	0.465

*MMSE, Mini-Mental State Exam; MoCA-B, Montreal Cognitive Assessment—Basic.*

*^a^Student’s t-test or Mann-Whitney U-test.*

*^b^χ^2^-test.*

Global amyloid standardized uptake value ratio (SUVR, uptake in whole gray matter/bilateral cerebellum crus) was available for 222 participants in all five groups. We next estimate the relationship between urine formaldehyde level and SUVR. However, we found no significant correlation between urine formaldehyde concentration and SUVR (Figure 3B).

### Correlation Analysis of Alzheimer’s Disease Plasma Biomarkers and Urine Formaldehyde Level

**FIGURE 4:**
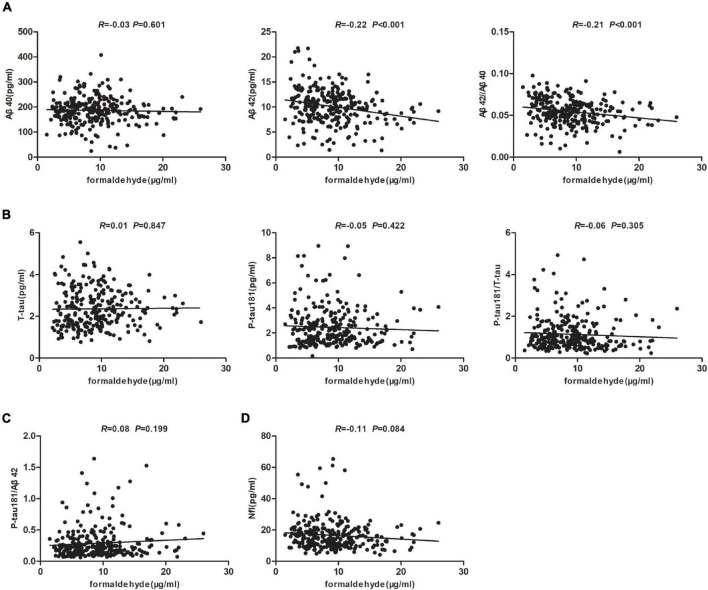
Correlation analysis of AD plasma biomarkers and urine formaldehyde level. **(A)** The correlations between urine formaldehyde and plasma Aβ40, Aβ42, Aβ42/Aβ40. **(B)** The correlations between urine formaldehyde and plasma T-tau, P-tau181, P-tau181/T-tau. **(C)** The correlations between urine formaldehyde and plasma P-tau181/Aβ42. **(D)** The correlations between urine formaldehyde and plasma NfL.

In order to clarify whether there is a correlation between plasma markers and urine formaldehyde in AD spectrum, we tested the blood biomarkers of AD pathology (Aβ40, Aβ42, T-tau, and P-tau181) and axonal injury (NfL) by Simoa assays. Plasma biomarkers were available for 267 participants in all five groups. We carried out correlation analysis to determine whether urine formaldehyde level was related to AD plasma biomarkers. The data indicated that urine formaldehyde level were negatively correlated with plasma Aβ42 level (*R* = −0.22, *P* < 0.001) and Aβ42/Aβ40 ratio (*R* = −0.21, *P* < 0.001) (Figure 4A). There was no correlation of plasma Aβ40 (Figure 4A), T-tau, P-tau181, P-tau181/T-tau (Figure 4B), P-tau181/Aβ42 (Figure 4C) and NfL (Figure 4D) with the concentration of urine formaldehyde.

In order to ensure that the correlations were not due to the effects of covariates, variables age, gender, education, APOE genotype, MMSE score, MoCA-B, plasma Aβ40, Aβ42, T-tau and P-tau181, Aβ42/Aβ40, P-tau181/T-tau and P-tau181/Aβ42 were added to the multiple linear regression model with the urine formaldehyde level as the dependent variable. The urine formaldehyde level showed significant independent correlation with gender, plasma Aβ42 and P-tau181/T-tau (Table 4).

**TABLE 4:** Multiple linear regression models with the urinary formaldehyde level as a dependent variable^a^.

Significant independent variables^b^	B	SE	Beta	*t*	*p*-value
Gender^c^	–2.593	0.654	–0.269	–3.963	0.000
Aβ42	–0.369	0.094	–0.270	–3.920	0.000
pTau181/Tau	–1.045	0.515	–0.140	–2.030	0.044

*^a^The data were determined by using stepwise regression analysis. (Adjusted R^2^ = 0.133, P = 0.000).*

*^b^Independent variables included in the analysis are as follows: age, gender, education, APOE genotype, Mini-Mental State Exam score, Montreal Cognitive Assessment—Basic score, plasma Aβ40, Aβ42, T-tau and P-tau181, Aβ42/Aβ40, pTau181/Tau, pT181/Aβ42.*

*^c^Gender ratio: male = 1, female = 2.*

## Discussion

AD is now considered a neurodegenerative disease with a very long progression that starts silently decades before the onset of symptoms and advances gradually until it compromises the person’s cognition. Therefore, the focus has been shifted from the clinical symptoms to the biological alterations and how to change the possibilities of future diagnosis and treatment.

Urine has become a highly desirable source of AD biomarkers, as it can easily and non-invasively be collected in relatively large volumes. Additionally, it contains cellular components, biochemical compounds, and proteins originating from plasma glomerular filtration, renal tubule excretion or urogenital tract secretion, thus reflecting the metabolic and pathophysiological condition of an individual (Balasa et al., 2020). The study of guide molecules of brain oxidative stress damage might be promising early hallmarks of AD in urine. Some metabolites such as the isoprostane 8,12-iso-iPF(2alpha)-VI, a free amino acid generated by lipid peroxidation whose levels in urine were higher at advanced AD stages, might predict the progression of the pathology from MCI to AD dementia (Praticò et al., 2002; García-Blanco et al., 2018). Other promising markers are some proteins found in urine, such as Alzheimer-associated neuronal thread protein (AD7c-NTP). Urine AD7c-NTP was increased in MCI and AD (Youn et al., 2011; Ma et al., 2015). However, AD7c-NTP was not elevated in patients with SCD (Li et al., 2019). While there have been considerable advancements in the field, the lack of changes on the above-mentioned markers in the preclinical stage of AD poses significant challenges for the use of such biomarkers in the clinical practice. In this study we focus on the identification of urine biomarkers, which could better reflect the preclinical changes of AD and be used as an available clinical strategy for the accurate and early detection of AD.

The current study made three major findings. Firstly, we revealed the relationship between urine formaldehyde and cognitive impairment of SCD, Obj-SCD, MCI, and AD patients. Urine formaldehyde level was expressed differently in different stages of AD. To be specific, the level of urine formaldehyde in patients with SCD was higher than that of cognitively normal people, while that in Obj-SCD and MCI patients was lower than that of SCD patients. In addition, the level of urine formaldehyde was significantly higher in AD patients compared to both NC and MCI groups. To our knowledge, this work is the first to show the dynamic variation characteristics of urine formaldehyde and reveal the correlation between urine formaldehyde and cognitive abilities throughout the AD continuum. In particular, abnormalities in urine-derived formaldehyde have been observed in SCD stage before objective cognitive decline. Though Aβ accumulation is the earliest pathological change which happens years before the clinical onset of AD (Aisen et al., 2017), Aβ detection by PET imaging or CSF is not universally available for patients screening or early diagnosis. Urine formaldehyde detection combined with comprehensive cognitive evaluation could be used as a tool to distinguish SCD population from NC and Obj-SCD population, also to discriminate MCI population from AD population. Secondly, we studied the effects of carrying APOE ε4 gene and cerebral Aβ accumulation on urine formaldehyde level. In analysis of these gender-and age-matched samples, our preliminary results suggested that the level of urine formaldehyde was higher in APOE ε4+ subgroup compared to APOE ε4- subgroup in both NC and AD groups, and there was no difference in urine formaldehyde level between the Aβ+ and Aβ- subjects in each group. Thirdly, we revealed the factors related to urine formaldehyde levels in AD spectrum, and found that after excluding the influence of covariates, urine formaldehyde level was significant independent correlated with gender, plasma Aβ42 and P-tau181/T-tau according to a multiple linear regression model. This further indirectly reflected the correlation between urine formaldehyde level and the disease severity of AD. As urine contains very little residual proteins, the formaldehyde level in urine probably reflect the status of endogenous formaldehyde metabolism. Excessive exposure to formaldehyde was reported to induce amyloid aggregation (Perna et al., 2001) and tau protein aggregation and hyperphosphorylation *in vitro and in vivo* (Lu J. et al., 2013). Mean-while, excess endogenous formaldehyde can lead to induces AD-like pathologies and cognitive impairments in rodent studies and rhesus monkeys (Yang et al., 2014; Zhai et al., 2018). Moreover, formaldehyde accumulated is associated with impaired performance in learning and memory in AD patients (Perna et al., 2001; Tong et al., 2011, 2017). Similarly, increased formaldehyde levels have also been observed in the hippocampus of AD patients (Tong et al., 2011). Taken together, these findings suggest that formaldehyde toxicity can be closely related to the critical hallmarks of AD pathology. The elevation of formaldehyde within the body may play an important role in AD development involving multifarious mechanisms. However, direct causal links and interconnected mechanisms remain to be fully demonstrated. In this study we had found that detection of the urine formaldehyde concentration was a feasible, non-invasive, and economical approach for predicting a cognitive deterioration and monitoring the degree of cognitive impairment in AD, which might facilitate the early diagnosis of the disease.

The SCD patients with SCD, but no significant deficits in the neuropsychological evaluation, appeared to be a group of particular interest for the early detection of a neurodegenerative disease. Those preclinical subjects without a remarkable neuropathy showed higher urine formaldehyde level than those prodromal subjects. We preliminary speculate that a more pronounced neuronal damage is needed to expose any signs of cognitive impairment, because of the more effective connections and the neuronal pathway usages in those particular cases. When the disease progresses to prodromal stage, some unknown protective factors lead to the compensatory decrease, and finally the body decompensates in AD dementia stage. However, the above conjecture needs further clinical studies and basic research to confirm. Besides the dominant amyloid cascade model, additional models of pathogenesis in AD include those highlighting the roles of endosomal recycling deficiency (Small and Petsko, 2020), immunity, lipid metabolism, endocytosis deficiency (van der Kant et al., 2020), and vascular dysfunction (Reitz and Mayeux, 2014). The dynamic concentration change of urine formaldehyde further supports the underlying AD pathogenic mechanism called formaldehyde stress may be present. Formaldehyde stress refers to the metabolic response, abnormal modifications of cellular proteins, protein misfolding, nuclear translocation and even cell death induced by excess extracellular and intracellular formaldehyde. Chronic impairments of the brain resulted from formaldehyde stress could lead to dysfunction in cognition such as learning decline and memory loss, and be one of the mechanisms involved in the process of senile dementia during aging (He et al., 2010; Wang et al., 2019).

This study has had some limitations, as expected. One of them was a cross-sectional design, which does not allow for causal interpretations and restricts the predictability of the urine formaldehyde values to those of a current cognitive performance. A longitudinal (long term follow-up) study is required to confirm whether there is indeed a causal relationship between the change in the urine formaldehyde level and the degree of cognitive impairment during the progression of AD. Also, the subjects were recruited from the memory clinic and limited by the small sample size, which makes a generalization of the whole population less reliable. The results of our cross-sectional study are preliminary and need further confirmation in longitudinal studies with larger samples.

Urine formaldehyde was found to be higher for individuals with subjective cognitive complaints and patients with MCI and AD dementia, suggesting that urine formaldehyde could be used as a biomarker for early diagnosis before objective cognitive decline and differential diagnosis at different stages of AD. Urine formaldehyde level were correlated with APOE genotype in NC and AD individuals, but were not correlated with brain Aβ deposition. In addition, after excluding the influence of covariates, urine formaldehyde concentration showed significant independent correlation with gender, plasma Aβ42 and P-tau181/T-tau. Determining the effective and specific biomarkers can help identify asymptomatic patients and estimating the risk of AD developing. This will not only allow for large-scale preliminary screening and early diagnosis in population, but also contribute to selection of suitable enrollees with a high risk of developing AD before the stage of dementia for further clinical trials. In the near future, the detection of formaldehyde in urine may be of key importance in monitoring the change in AD and reflecting the therapeutic effect. In addition, deep exploration of the urine metabolome for biomarkers relevant to AD may yield valuable mechanistic information for AD pathology and created a new concept of the disease.

## Data Availability Statement

The raw data supporting the conclusions of this article will be made available by the authors, without undue reservation.

## Ethics Statement

The studies involving human participants were reviewed and approved by the Shanghai Jiao Tong University Affiliated Sixth People’s Hospital. The patients/participants provided their written informed consent to participate in this study.

## Author Contributions

QG contributed to the study design, research subjects’ recruitment. RH contributed to the study design and urine formaldehyde concentration detection. YW contributed to the analyses of data, interpretation of data, and creating and revising the content. FP contributed to the collection of data and revised the manuscript for content. FX contributed to the analyses of PET image and revised the manuscript for content. All authors contributed to the article and approved the submitted version.

## Conflict of Interest

The authors declare that the research was conducted in the absence of any commercial or financial relationships that could be construed as a potential conflict of interest.

## Publisher’s Note

All claims expressed in this article are solely those of the authors and do not necessarily represent those of their affiliated organizations, or those of the publisher, the editors and the reviewers. Any product that may be evaluated in this article, or claim that may be made by its manufacturer, is not guaranteed or endorsed by the publisher.
